# Effects and mechanisms of acupuncture for PIGD-subtype Parkinson’s disease via integration of fMRI and gut microbiota-metabolomics analysis: protocol for a prospective randomized controlled trial

**DOI:** 10.3389/fnagi.2025.1534165

**Published:** 2025-05-13

**Authors:** Jili Sheng, Yingqi Xu, Tao Liu, Jianfang Zhu, Caiyun Mu, Lihao Zhai, Shanhu Xu, Hanyi Wang, Xiangzhi Liu, Tao Liu, Xiaoqing Jin

**Affiliations:** ^1^Department of Acupuncture, Zhejiang Hospital, Hangzhou, China; ^2^The Second Clinical Medical College of Zhejiang Chinese Medical University, Hangzhou, China; ^3^Department of Radiology, Zhejiang Hospital, Hangzhou, China; ^4^Department of Neurology, Zhejiang Hospital, Hangzhou, China; ^5^The State Key Laboratory of Fluid Power and Mechatronic Systems, School of Mechanical Engineering, Zhejiang University, Hangzhou, China

**Keywords:** Parkinson’s disease, acupuncture, fMRI, intestinal flora, metabolomics, randomized controlled trial, protocol

## Abstract

**Introduction:**

Parkinson’s disease (PD) can be categorized into various subtypes based on the primary symptoms associated with motor dysfunction. One subtype, known as postural instability and gait difficulty (PIGD), is characterized by severe clinical symptoms, an increased risk of walking difficulties and falls, and a poorer prognosis compared to other subtypes. This condition imposes a significant burden on patients, their families, and the healthcare system. Recently, acupuncture, a practice rooted in traditional Chinese medicine, has gained attention for its potential to influence neurophysiological pathways and enhance the overall brain function in individuals with PD. This randomized controlled study aimed to evaluate the clinical effectiveness of acupuncture in patients with the PIGD subtype of PD and to investigate the preliminary exploration of mechanisms of acupuncture by analyzing intestinal microbiota and metabolomics, thereby providing deeper insights into its impact on patients.

**Methods:**

This randomized controlled trial will involve 64 patients diagnosed with the PIGD subtype of PD. Participants in both groups will undergo three acupuncture sessions weekly for a duration of 4 weeks, followed by an 8-week follow-up period. The primary outcome measure will be the Unified Parkinson’s Disease Rating Scale III. Secondary outcomes will include the Berg Balance Scale (BBS), wearable gait analysis, and functional magnetic resonance imaging (fMRI). Additionally, serum and stool samples will be collected for 16S ribosomal RNA sequencing, and liquid chromatography coupled with tandem mass spectrometry analysis (LC–MS/MS) will be employed to elucidate theunderlying mechanisms. This trial has been reviewed and approved by the Medical Ethics Committee of Zhejiang Hospital (Approval no. 2023-15 K). Participation in this study will require written informed consent from all patients. The findings of this study will be published in a peer-reviewed journal, and there will be no restrictions on publication.

**Discussion:**

In this study, we integrate traditional assessment scales with fMRI to demonstrate the therapeutic effects of acupuncture. We will also analyze the modulation of gut microbiota and serum metabolome to explore the underlying neural mechanisms. Our results will provide a foundation for future studies in this area.

**Clinical trial registration:**

https://www.chictr.org.cn, identifier ChiCTR2300071703.

## 1 Introduction

Parkinson’s disease (PD) is a prevalent neurodegenerative disorder resulting from a complex interaction of genetic, environmental, and lifestyle factors (Poewe et al., 2017). There are various clinical manifestations of PD, including tremor-dominant (TD), postural instability and gait difficulties (PIGD), and the mixed type. Among these, the PIGD subtype presents particular challenges, as it often leads to significant motor impairment and a decreased quality of life for both patients and their families (Stebbins et al., 2013; Hattori, 2016; Tysnes and Storstein, 2017). Patients with the PIGD subtype are more susceptible to severe symptoms, frequent falls, and poorer prognosis than those with the TD subtype, thereby increasing the burden on caregivers and the healthcare system (Pelicioni et al., 2018; Pelicioni et al., 2019). Current treatment strategies often yield suboptimal results for the PIGD subtype; hence, complementary approaches such as acupuncture are suggested to potentially alleviate PD symptoms and improve patients’ overall wellbeing (Ahrweiller et al., 2019; Shin et al., 2020).

Acupuncture, a traditional Chinese practice, has demonstrated potential benefits in alleviating both motor and non-motor symptoms of PD (Xu et al., 2020; Lei et al., 2023; Li et al., 2023). In contrast to previous studies that primarily relied on scale recordings, our trial will employ advanced methodologies, including wearable gait analysis and functional magnetic resonance imaging (fMRI) assessments, alongside a multimodal approach to understanding the underlying mechanisms. These methodologies will be used to clarify how acupuncture enhances gait and brain function and explore its preliminary mechanisms of action and clinical benefits, particularly in individuals with the PIGD subtype.

Recent studies have indicated that the gut-brain axis significantly influences PD pathology and correlates with disease severity (Liu et al., 2024). Acupuncture has been employed to enhance brain function and mitigate inflammation in neurological diseases including PD, while also addressing gastrointestinal dysfunction in various gastrointestinal disorders (Zhang et al., 2023). By investigating the gut microbiome and its metabolites, researchers can gain insights into how acupuncture impacts the gut-brain axis and PD pathology. This comprehensive approach could provide a thorough understanding of the therapeutic effects of acupuncture and identify potential biomarkers or targets for future interventions (Jia et al., 2012; Fathi et al., 2013).

In summary, we will conduct a randomized clinical trial to evaluate the safety and efficacy of acupuncture as an adjunct treatment for patients with PIGD subtype of PD. Additionally, we will preliminarily explore the mechanisms underlying the therapeutic effect of acupuncture from the perspective of the gut flora-metabolite axis, which will establish a foundation for future research.

## 2 Methods and analysis

### 2.1 Study design

The study will be a single-center, prospective, randomized controlled trial. The Medical Ethics Committee of Zhejiang Hospital (Hangzhou, China; Approval no. 2023-15K) reviewed and approved the protocol in May 2023, and it was registered at www.chictr.org.cn (ChiCTR2300071703). The study design adheres to the Standard Protocol Items Recommendations for Interventional Trials (SPIRIT).

### 2.2 Recruitment strategy and consent

**Figure 1:**
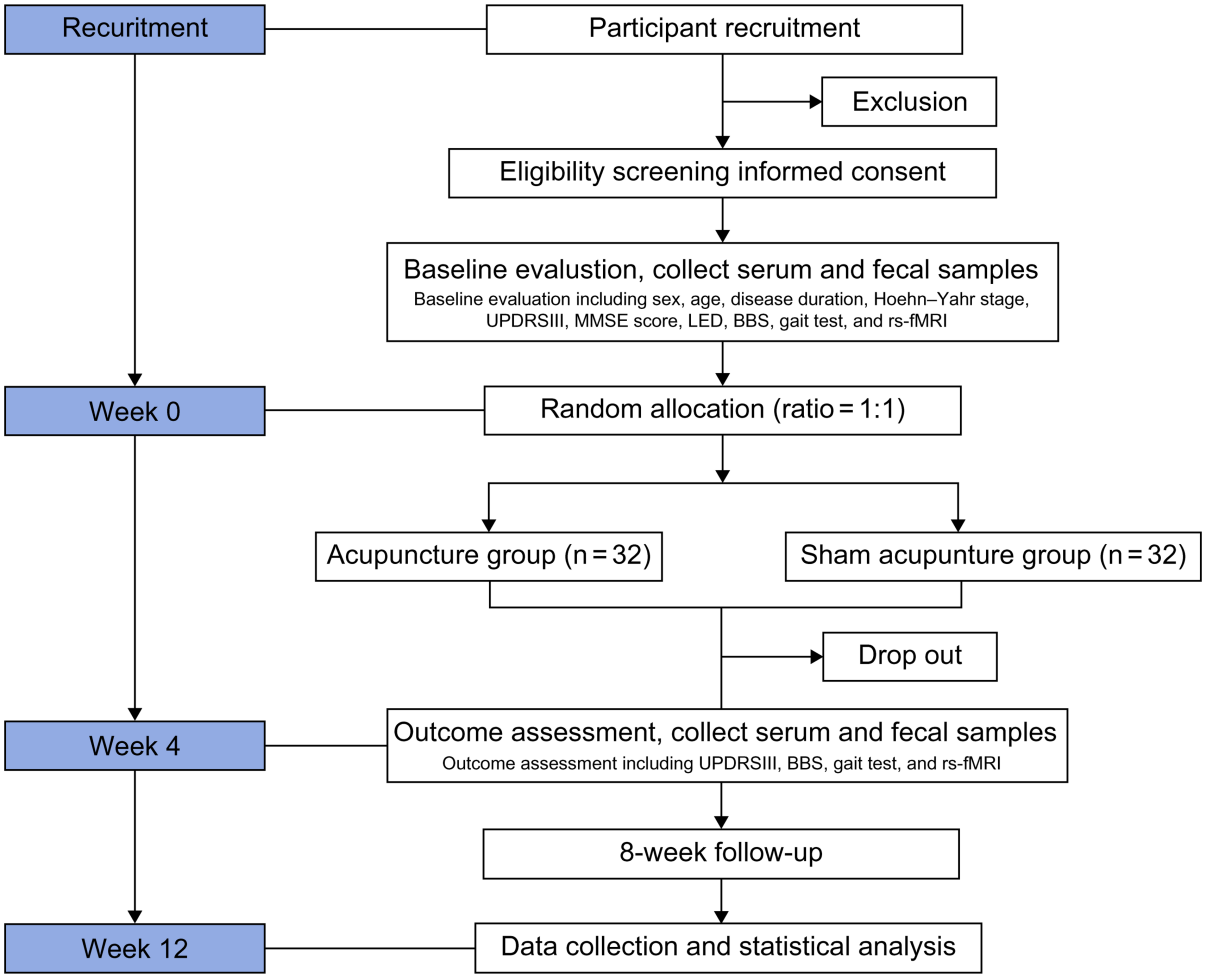
Flowchart depicting the study protocol. MMSE, Mini-Mental State Examination; UPDRSIII, Unified Parkinson’s Disease Rating Scale III; LED, levodopa equivalent dose; BBS, Berg Balance Scale; rs-fMRI, resting-state functional magnetic resonance imaging.

Eligible participants will complete the entire investigation at Zhejiang Hospital. A clinical researcher will thoroughly explain the study objectives and requirements to participants before inclusion. This process ensures that participants have a clearly understanding of the study, and only those who provide written informed consent, personally or through their legal representatives, will be included in the baseline assessment. The trial will consist of a baseline assessment period of approximately lasting 1 week, a 4-week intervention period, and an eight-week follow-up period. Figure 1 illustrates the study flowchart, while Table 1 presents the detailed schedule for participant enrollment, treatments, and assessments.

**Table 1:** Enrollment, treatment, and assessment schedule.

Period of study	Screening	Baseline week 0	Treatment week 4	Follow-up week 12
Screening of applicants (based on inclusion and exclusion criteria)	✓			
Demographic data	✓			
Baseline evaluation				
• Disease duration	✓			
• Hoehn–Yahr scale	✓			
• MMSE	✓			
• Moca	✓			
• LED	✓			
Informed consent	✓			
Treatment		✓	✓	
Outcome assessment				
• UPDRSIII		✓	✓	✓
• BBS		✓	✓	✓
• Gait analysis		✓	✓	✓
• rs-fMRI		✓	✓	
Sample collection				
• Serum		✓	✓	
• Fecal		✓	✓	
Safety assessment		✓	✓	✓

✓: required; MMSE, Mini-Mental State Examination; UPDRSIII, Unified Parkinson’s Disease Rating Scale III; LED, levodopa equivalent dose; BBS, Berg Balance Scale; rs-fMRI, resting-state functional magnetic resonance imaging.

### 2.3 Diagnostic criteria

The diagnostic criteria will adhere to the standards established by the International Movement Disorder Society in 2015 (Berg et al., 2015). Specifically, for the PIGD subtype, the criteria will be fulfilled if the ratio of the mean scores for these disturbances to the tremor score is ≤0.90 (Stebbins et al., 2013).

### 2.4 Recruitment criteria

The following inclusion criteria will be utilized:

(1) A definitive diagnosis of primary Parkinson’s disease according to the standards set by the UK Parkinson’s Disease Society Brain Bank (Demonceau et al., 2015; Hughes et al., 1992). (2) A Hoehn-Yahr level ranging from 1.5 to 3.0 based on the PD grading scale. (3) No restrictions based on sex. The participants must be right-handed and aged between 50 and 85 years (Right-handedness is a standard requirement in fMRI research to ensure consistency in brain hemisphere activation and to minimize confounding variables related to handedness.) (4) Adherence to the western medicine treatment plan for a duration of 3 months or longer, while excluding the use of antibiotics, probiotics, anti-inflammatory drugs, excretory medications, immunosuppressants, acid–base balance medications, and any physical or traditional Chinese medicine treatments (To enhance feasibility, patients were permitted to transition gradually to non-restricted medications under physician supervision before enrollment). (5) The Mini-Mental State Examination (MMSE) and Montreal Cognitive Assessment (MoCA) must indicate that participants exhibit no cognitive impairment. (6) Normal vision, hearing, and language abilities. (7) Participants must provide informed consent and willingly agree to participate.

### 2.5 Exclusion criteria

Participants will be excluded from the study if they meet any of the following conditions:

(1) A diagnosis of secondary Parkinson’s syndrome or cognitive impairment. (2) Severe hypertension, vascular disease, cardiac insufficiency, or systemic bone and joint diseases that impair walking ability. (3) Contraindications for undergoing fMRI. (4) Receipt of medications such as antibiotics, probiotics, anti-inflammatory drugs, excretory drugs, immunosuppressants, acid–base balance medications, and traditional Chinese medicine within 3 months prior to enrollment (Dethlefsen and Relman, 2011; Palleja et al., 2018). (5) Concurrently participating in other research.

### 2.6 Dropout criteria

Participants will be considered to have dropped out of the study if they meet any of the following conditions:

(1) Patients who voluntarily withdraw or do not complete the trial as planned. (2) Inability to continue the study due to adverse events. (3) Patients who discontinue the study early.

### 2.7 Termination criteria

The experiment will be terminated if the following conditions are met:

(1) Modifications to PD drug doses. (2) Probiotics and other physical or traditional Chinese medicine treatments are employed throughout the trial. (3) Inability to continue the trial due to serious adverse events. (4) Disease deterioration or serious complications during the trial. (5) Patients are unable to participate in the trial due to other uncontrollable factors.

### 2.8 Randomization, allocation concealment, and blinding

Participants will be divided into two groups: the acupuncture group (receiving acupuncture treatment) and the sham acupuncture group (receiving pseudo-acupuncture treatment). The allocation ratio is set to 1:1 (*n* = 64), with randomization executed using a random group number generator in SPSS software (IBM, Armonk, NY, USA). Random numbers will be placed in opaque envelopes, which will be sealed and labeled with corresponding serial numbers to ensure confidentiality. The envelopes will be opened sequentially, and participants will be assigned to their respective treatment groups.

While patients in this trial will be blinded, it is nearly impossible to blind the practitioners and operators since they must know which acupuncture devices to use. They are prohibited from discussing the acupuncture devices or the study’s purpose with participants. Operators and assessors will be kept separate, and data analysis will be performed by an independent team. Outcome assessors and statisticians will be blinded to the treatment distribution.

To evaluate the success of blinding, patients will complete a blinding validation questionnaire (BTQ) immediately after their treatment. The BTQ consists of five questions. Blinding is considered successful if a participant completes all five questions without hesitation, reports no unusual sensations, and does not deny receiving acupuncture. Conversely, blinding is considered unsuccessful if participants identify any unusual elements of the questionnaire or deny receiving acupuncture. Otherwise, it is deemed successful.

### 2.9 Sample size estimation

The sample size was determined based on the Unified Parkinson’s Disease Rating Scale (UPDRS) III motor assessment. A previous study (Jang et al., 2020a) provided the sample size estimation. Two-sided two-sample *t-*tests with a significance level of 0.05 were employed to achieve 80% power. The analysis indicated a standard deviation of 1.54 and a mean difference of 1.15 between the two groups. Consequently, the G-Power (v3.1) sample size calculation indicated that each group will consist of 29 patients diagnosed with PIGD-subtype PD, satisfying the inclusion and exclusion criteria. However, to accommodate a 10% attrition rate, the sample size for each group was increased to 32 patients.

### 2.10 Intervention

Patients will receive standard medical treatment as outlined in the Parkinson’s Treatment guidelines (Pringsheim et al., 2021). This study includes both acupuncture and sham manipulation. The acupuncture group will receive an additional 30 min of acupuncture treatment. All acupuncture treatments will be performed by two senior physicians with over 5 years of clinical experience, administered three times weekly for 4 weeks.

### 2.11 Acupuncture points

**Figure 2:**
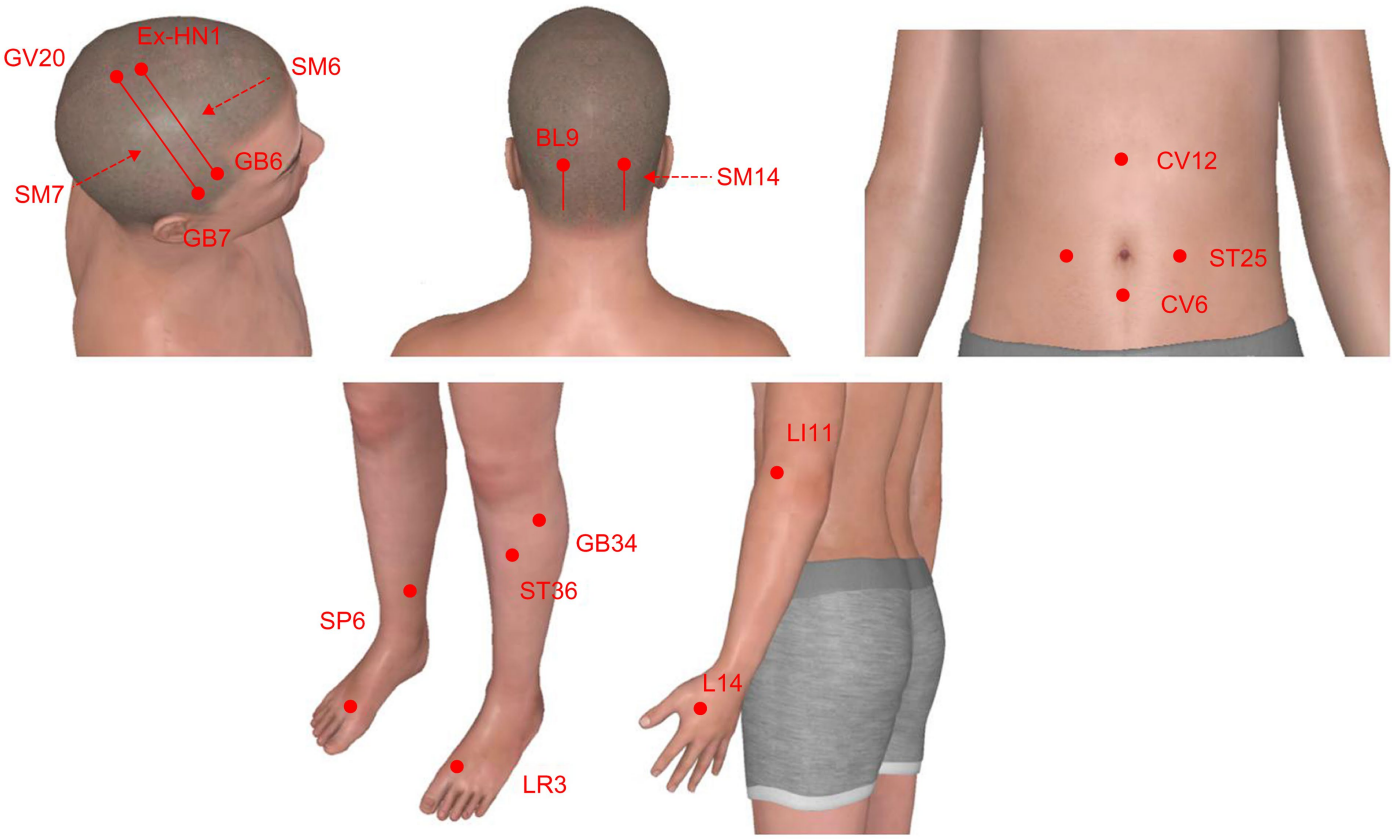
Locations of scalp acupuncture lines and acupoints. Mannequin images were sourced from the 3D body application (v8.8.32).

The acupuncture points selected include the upper 1/5 anterior oblique line of the vertex-temporal (MS6), the upper 1/5 posterior oblique line of the vertex-temporal (MS7), and the lower-lateral line of the occiput (MS14), according to the proposed standard international acupuncture nomenclature proposed by the WHO scientific group in 2020 (Wise and Lorenc, 2023). Additional acupuncture points include Hegu (LI4), Quchi (LI11), Taichong (LR3), Yanglingquan (GB34), Zusanli (ST36), Sanyinjiao (SP6), Zhongwan (CV12), Tianshu (ST25), and Qihai (CV6), following the standards set by the standards of The People’s Republic of China, GB/T12346-2006, 2006. Details of the selected acupoints are presented in Table 2 and Figure 2.

**Table 2:** Locations of scalp acupuncture lines and acupoints.

Points	Location
Anterior oblique line of vertex-temporal (MS6)	Located on the side of the top of the head, the line 1 cun anterior to Qianshenchong (one of the four acupuncture points collectively designated as Ex-HN1) obliquely to Xuanli (GB6).
Posterior oblique line of vertex-temporal (MS7)	Located on the side of the top of the head, 1 cun after the anterior oblique line of the parietal and temporal, and parallel to it, that is, the line from Baihui (GV20) obliquely to Qubin (GB7).
Lower-lateral line of the occiput (MS14)	Located in the occipital region, a line 2 cun long from Yuzhen (BL9) straight down.
Hegu (LI4)	Located on the back of the hand, between the first and second metacarpals, at the midpoint of the second metacarpal.
Taichong (LR3)	Located on the dorsum of the foot, between the 1st and 2nd metatarsal bones, in the anterior depression at the junction of the metatarsal base, or where the pulse can be felt.
Quchi (LI11)	Located at the elbow, specifically at the midpoint between the Qiuze (PC3) and the external epicondyle of the humerus.
Yanglingquan (GB34)	Located on the lateral side of the lower leg, in the depression near the anterior border of the fibula.
Zusanli (ST36)	Located on the lower leg, approximately 3 cun below ST 35 and one finger-width lateral to the anterior border of the tibia.
Sanyinjiao (SP6)	Located on the inner aspect of the lower leg, about 3 cun above the prominent bone on the inner ankle
Zhongwan (CV12)	Located on the midline of the abdomen, directly above the navel, approximately 4 cun below the sternal xiphoid process.
Tianshu (ST25)	Located on the midline of the abdomen, 2 cun lateral to the navel on both sides.
Qihai (CV6)	Located on the midline of the abdomen, approximately one and a half finger-widths below the navel.

### 2.12 Acupuncture operation

During the operation, participants will be instructed to wear eye masks and perform the head stitch while seated. First, the location of the scalp acupoint will be identified, and the tip of the needle will be inserted diagonally below the subcutaneous fascia along the acupoint area from top to bottom. The needle should be positioned at an angle of 15° to 30° relative to the scalp, with the tip from the top down into the subcutaneous fascia and then advanced 0.5 to1.0 cun along the scalp acupoint line.

Participants will be then be instructed to perform body acupuncture procedures in a supine position. Once their breathing is stable and their bodies are fully relaxed, the following steps will be performed: The needle will be inserted straight into the acupoints LI4, SP6, and LR3. For LI4 and SP6, the insertion depth should range from 0.5 to 0.8 cun, whereas for LR3, the depth should range from 0.3 to 0.5 cun. The needles should be inserted at an angle of 15° to 30° from the skin surface to ensure proper stimulation.

Additionally, a straight insertion will be performed into LI11, CV12, ST25, and CV6. The insertion depth for LI11 and CV12 should be between 1.0 and 2.5 cun, while for ST25 and CV6, it should be between 0.8 and 1.0 cun. The needles should be inserted perpendicularly to the skin at a 90° angle.

Finally, the needle will be inserted from the outer side of the calf toward GB34 and ST36 at a 90° angle, either straight or obliquely, to a depth of 1.5 cun. The insertion should be done carefully to avoid discomfort. After insertion, the needles at LI11, CV12, ST25, CV6, ST36, and GB34 can be lifted and twisted gently to enhance stimulation, generating a local swelling sensation that indicates the achievement of deqi. In Traditional Chinese Medicine (TCM), deqi is a key concept in acupuncture and moxibustion, signifying the achievement of an optimal therapeutic state during the course of treatment (MacPherson and Asghar, 2006; Zhou et al., 2011).

**Figure 3:**
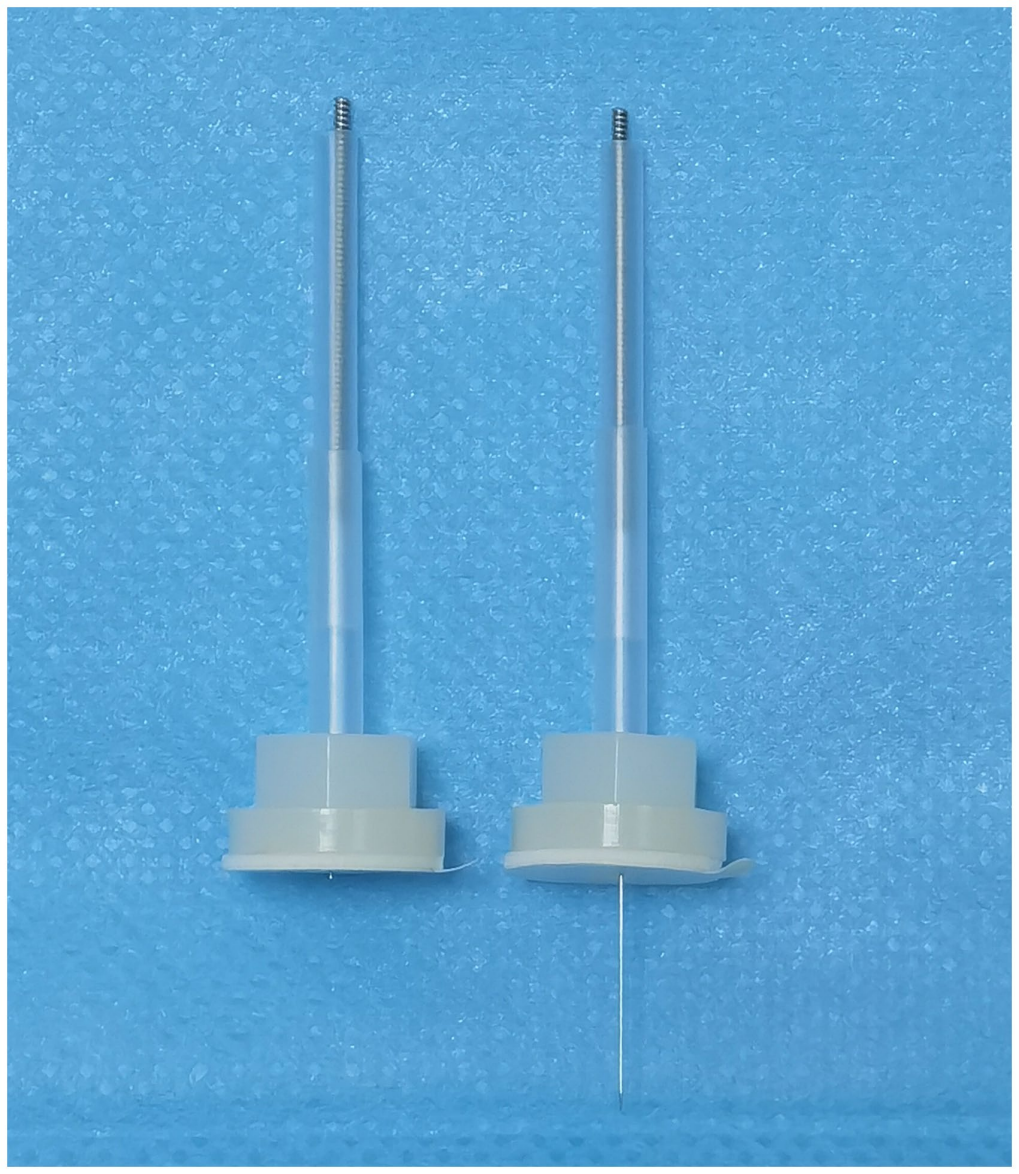
Device for acupuncture treatment and fixation. The device on the left is for the sham acupuncture group and on the right for the acupuncture group. Sham needles (blunt-tip design, left) were affixed with elastic fixation devices to mimic tactile stimulation without skin penetration.

During the procedure, sterile disposable filament needles, measuring between 25 and 40 mm in length and 0.25 mm in diameter will be utilized, along with appropriate fixation tools (details are presented in Figure 3). The needles are manufactured by Suzhou Huatuo Medical Equipment Company in China to ensure quality and safety.

All operators will undergo standardized training to adhere to the specified needle insertion angle, depth, and stimulation intensity. This training will encompass practical demonstrations and assessments to ensure consistent practice among practitioners, supplemented by regular skill evaluations and practice audits to maintain high standards of care.

### 2.13 Sham acupuncture operation

The acupoints utilized in the sham acupuncture group will mirror those of the acupuncture treatment group. The sham acupuncture group will utilize non-penetrating retractable blunt needles and fixation tools that do not penetrate, which also measure between 25 and 25 mm in length and 0.25 mm in diameter, sourced from the same manufacturer (details are presented in Figure 3). Patients in this group will wear the same eye masks as those in the acupuncture group. The sham procedure will employ the same number of needles and last the same duration as the acupuncture procedure. Acupuncture points on the head will be secured with tape, while those on the body will be stabilized using the device depicted in Figure 3. All operational details will be meticulously documented.

### 2.14 Baseline evaluation

During the baseline evaluation, the patient’s age, sex, disease duration, Hoehn–Yahr stage, UPDRS III scores, MMSE scores, MoCA scores, and levodopa equivalent dose (LED) will be assessed and recorded. To ensure the effectiveness of the study, assessments will be conducted at the same time each time the patient is in the on or off phase.

### 2.15 Outcome assessment

Throughout the observation period, each patient will record detailed information regarding their medication use, including dose modifications and interactions with acupuncture. All PD patients will begin the tests 12 h after discountinuing their anti-PD medication (the off period) and will take their medication immediately after completing the test.

#### 2.15.1 Primary outcomes

The UPDRS III assesses a patient’s motor function by measuring various aspects, such as language, facial expressions, tremors, rigidity, limb movements, posture, and gait. A lower UPDRS III score indicates a better quality of life (Movement Disorder Society Task Force on Rating Scales for Parkinson’s Disease, 2003). This evaluation will be performed prior to inclusion, 4 weeks post- treatment, and after 8 weeks of follow-up.

#### 2.15.2 Secondary outcomes

##### 2.15.2.1 The Berg balance scale

The Berg Balance Scale (BBS) is a widely utilized assessment tool that evaluates a patient’s capacity to maintain both dynamic and static balance through a series of functional activities (Park, 2018). Higher scores on this scale signify enhanced balance capability. This evaluation will be conducted prior to inclusion, after 4 weeks after treatment, and after 8 weeks of follow-up.

##### 2.15.2.2 Gait test

Wearable gait analysis systems employ sensors and computer systems for gait quantification. By combining motion sensors worn on both feet, the systems can capture the three-dimensional (3D) attitude angle and motion trajectory. Abnormal parameters associated with gait posture can be identified by measuring gait frequency and amplitude stability. Subsequently, the data from the sensors are analyzed to provide a comprehensive gait assessment (Demonceau et al., 2015; Nancy Jane et al., 2016). An inertial measurement unit (IMU) developed by the School of Mechanical Engineering at Zhejiang University will be utilized to analyze the gait of patients with PD (Wang et al., 2019). After the patient traverses a distance of 20–40 m on level ground, an IMU will be positioned 3–5 cm above the ankle, to record data at a frequency of 100 Hz. The data stored on the SD card will include the gait cycle, gait speed, gait frequency, maximum moving speed, proportion of swing period, and proportion of support period. This evaluation was conducted prior to inclusion, after 4 weeks after treatment, and again after 8 weeks of follow-up.

##### 2.15.2.3 Resting-state fMRI

Resting-state fMRI measures blood-oxygen-level-dependent (BOLD) signals without the requirement of specific tasks, providing insights into spontaneous brain activity. All patients will undergo scanning prior to inclusion and 4 weeks post-treatment. A Siemens MAGNETOM Skyra 3.0 T scanner will be utilized to acquire MRI images. To minimize head movement during data collection, all patients will be fitted with foam pads. Additionally, to mitigate scanning noise, patients will be instructed to close their eyes and refrain from thinking of anything other than falling asleep. Two functional images (rest1 and rest2) and 3D T1-weighted images will be acquired from the scan. The fMRI parameters will include 32 axial slices, a repetition time (TR) of 2,000 ms, a echo time (TE) of 30 ms, a slice thickness of 3.5 mm, a flip angle (FA) of 90°, a matrix size of 64 × 64, and a field of view (FOV) of 224 × 224 mm^2^. The scan parameters for the T1-weighted structural image will be as follows: 256 slices, TR = 2,530 ms, TE = 2.98 ms, thickness = 1.0 mm, FA = 7°, matrix = 256 × 256, and FOV = 256 × 256 mm^2^.

Data will be analyzed using RESTplus (v1.2) software (Jia et al., 2019). The data will be preprocessed through the following steps: (1) 10 time points will be removed at the begininning of the scan to ensure the stability in the patient’s condition and allow for adaptation. (2) Correction of slice timing for different slices within an image. (3) Rearranging scans over time. (4) Resampling to the Montreal Neurological Institute space with a voxel size of 3 × 3 × 3 mm^3^. (5) Spatial smoothing using a 6 mm full width at half maximum Gaussian kernel to reduce individual mismatches. (6) Linear trend removal. (7) White matter signals, Friston-24 head motion parameters, and cerebrospinal fluid signals will be included in covariate regression. (8) Low-pass filtering will be applied between the range of 0.01 to 0.08 during the regression (Dunn et al., 2011).

Several calculation indicators, including Regional Homogeneity (ReHo), the amplitude of low-frequency fluctuations (ALFF), and functional connectivity (FC), have been proposed to assess brain activity and are considered more reliable (Zang et al., 2004; Zou et al., 2008; Arbabshirani et al., 2013). ALFF reflects the level of spontaneous activity in each brain region, with abnormal alterations in ALFF values indicating atypical neural activity. Conversely, abnormal increases in ReHo values suggest enhanced consistency in local spontaneous activity. Additionally, FC aims to establish connections between different brain regions based on temporal correlations.

### 2.16 Mechanism exploration

#### 2.16.1 Fecal collection and 16S ribosomal RNA sequencing analysis

After enrollment, participants will be required to maintain a stable diet, which will be documented through a food and medication diary. During stool sample collection, bowel frequency, consistency according to the Bristol stool scale (Lewis and Heaton, 1997), and any recent changes in dietary habits will be simultaneously recorded.

Fresh fecal samples will be collected prior to inclusion and 4 weeks post-treatment. Using disposable gloves, a spoon will mix the fecal samples to ensure that they are approximately the size of a broad bean before being placed in a fecal collection box. After collection, the samples will be immediately checked and stored at −80°C. Microbial DNA will be extracted from the fecal samples using a DNA extraction kit (MagPure Stool DNA KF Kit B; MAGEN, Guangzhou, China). The extracted DNA will be amplified via polymerase chain reaction targeting the highly variable V3-V4 regions of the 16S ribosomal RNA gene. The forward primer (338F 5’-ACTCCTACGGGAGGCAGCAG-3′) and reverse primer (806R 5’-GGACTACHVGGGTWTCTAAT-3′) will be utilized to amplify the V3-V4 region of the bacterial 16S ribosomal RNA gene. The libraries will be sequenced using the Illumina MiSe platform (Beijing Genomics Institution, Shenzhen, China).

Following the filtering of the raw data, further analyzed will be conducted, including tag linking, clustering of operational taxonomic units (OTUs), OTU taxonomic annotation, diversity analysis, and functional prediction. Notably, Mothur (v1.31.2) will be employed for alpha diversity analysis, while QIIME (v1.80) will be utilized for beta diversity analysis (Schloss Schloss et al., 2009; Caporaso et al., 2010). Functional prediction will be performed using PICRUSt (v2.3.0-b) (Douglas et al., 2019), and heat maps for additional visualizations will be created using the R package (v3.5.1).

#### 2.16.2 Serum preparation and liquid chromatography coupled to tandem mass spectrometry analysis (LC–MS/MS)

Blood samples will be collected from the patients prior to inclusion and again 4 weeks post-treatment. The samples will be allowed to coagulate in a centrifuge tube for 30 min. After complete coagulation, the blood will be centrifuged at 1600 × *g* at low speed for 10 min at 4°C. To obtain the supernatant for LC–MS analysis, it will be transferred into a new Eppendorf tube and centrifuged for an additional 10 min at 16000 × *g*.

The separation and detection of metabolites will be performed using a Waters 2777c UPLC (Waters, USA) in conjunction with a Thermo Fisher Scientific Q Exactive HF mass spectrometer (Thermo Fisher Scientific, MA, USA). Chromatographic separation will be conducted using a Waters ACQUITY UPLC BEH C18 column (1.7 μm, 2.1 mm × 100 mm, Waters, USA), with the column temperature maintained at 45°C. In positive mode, the mobile phase will consist of 0.1% formic acid (A) and acetonitrile (B), while in negative mode, it will comprise 10 mM ammonium formate (A) and acetonitrile (B). The flow rate will be set to 0.35 mL/min, and the injection volume will be 5 μL. The Q Exactive HF (Thermo Fisher Scientific) will be utilized for primary and secondary mass spectrometry data acquisition. The full scan range will set to 70–1,050 m/z with a resolution of 120,000, and the automatic gain control target for mass spectrometry acquisitions will be set to 3e6 with a maximum ion injection time of 100 ms. The data acquisition system employed will be the Thermo Fisher Scientific Q Exactive HF, with the automatic gain control target for mass spectrometry also set to 3e6 and a maximum ion injection time of 100 ms (Dunn et al., 2011).

The BGI Metabolome Database (BMDB), mzCloud database, and ChemSpider online database will be utilized to analyze the mass spectrometry data. The results of this analysis will be compiled into a data matrix containing information such as peak areas and identification results for metabolites. The subsequent bioinformatics analysis will encompass data preprocessing, quality control, species annotation, and the identification and analysis of differences between comparison groups. Species annotation will be conducted using the Human Metabolome Database and the Kyoto Encyclopedia of Genes and Genomes (KEGG) databases. Metabolites will be functionally annotated in the KEGG PATHWAY database, focusing on biochemical metabolic and signal transduction pathways. Differences between comparison groups will be analyzed using partial least squares-discriminant analysis (PLS-DA), while a two-tailed Student’s *t*-test will be employed to determine the relative metabolite contents between groups. Metabolites with a variable importance (VIP) score >1 will considered to exhibit differential alterations between the groups.

#### 2.16.3 Multimodal data analysis

In this study, we will conduct a comprehensive multimodal data analysis by integrating fMRI metrics including ALFF, ReHo and FC, with gut microbiota alpha and beta diversity, as well as differential serum metabolite levels. To explore the associations among these variables, we will utilize Spearman correlation analysis, which is well-suited for assessing non-parametric relationships. The Bonferroni correction will be applied to adjust for multiple comparisons. Additionally, mediation analysis will be performed to validate the hypothesized “microbiota-metabolite-brain function” pathway, examining how gut microbiota may influence brain function via specific metabolites. This integrated approach aims to provide deeper insights into the complex interactions among these biological systems.

### 2.17 Data analysis methods

Patient demographics, clinical characteristics, and gait data will be analyzed using SPSS V27.0. Measurement data will be expressed as mean ± standard deviation (mean ± SD) if they follow a normal distribution, while count data will be described using frequency and constituent ratio if they do not. For non-normally distributed data, the difference between the lower and upper quartiles (P25, P75) will be reported. Wilcoxon rank sum and Mann -Whitney U tests will be used for comparisons within and between groups. The chi-square test will be employed to analyze count data. Based on the normality of the data, either Pearson or Spearman correlation will be performed to analyze the correlations. This study will be deemed statistically significant at *p* < 0.05.

### 2.18 Data management and quality control

The research team is responsible for data management in this study to ensure the authenticity, integrity, privacy, and traceability of the data. All phases of data processing phases will adhere to standard operating procedures for quality control, thereby ensuring the accuracy and reliability of the original data obtained from the clinical trial. Additionally, any modifications to the case report form will be documented along with the reasons for these changes.

### 2.19 Trial supervision committee

A Trial Supervision Committee (TSC) will oversee the trial’s progress. Principal investigators will provide reports to the TSC biannually through written documentation. Adverse events will be meticulously recorded and managed promptly. Potential symptoms including syncope, subcutaneous hematoma, severe pain, dizziness, and bent or stuck needles, will be monitored at each session as indicators of the intervention’s safety. A ppropriate measures will be implemented to mitigate these risks. While this is classified as a low-risk level, severe adverse events although unlikely will be thoroughly reviewed by the TSC and ethics committees if they occur. Follow-up will be conducted for all adverse events until resolution.

## 3 Discussion

PD is an increasingly prevalent neurodegenerative disorder characterized both motor and non-motor symptoms, largely impacting patients’ quality of life. Among the various of PD subtypes, the PIGD subtype presents a considerable challenge due to its association with severe symptoms and an increased risk of falling (Pelicioni et al., 2018; Pelicioni et al., 2019). Thererfore, complementary treatments, such as acupuncture, may be beneficial in alleviating symptoms and enhance overall wellbeing (Deuschl et al., 2022).

Evidence suggests that acupuncture can enhance motor function, particularly gait and balance function in patients with PD (Fukuda et al., 2015; Pereira et al., 2021). However, most studies have treated PD as a homogeneous group, with no subtype analyses conducted. Notably, this research addresses a critical gap in the literature, as there is a scarcity of studies focusing specifically on the effects of acupuncture on PIGD subtype.

From a Traditional Chinese Medicine (TCM) perspective, the PIGD subtype of PD is associated with qi stagnation due to Yin deficiency in the liver and kidney, alongside deficiencies in qi and blood. These imbalances negatively affect organ function and meridian patency. Targeted acupuncture at specific points is postulated to regulate qi and blood flow, ultimately improving symptoms. Acupoints such as Yanglingquan (GB34) and Zusanli (ST36) are known to enhance qi circulation and have distinct physiological roles that aid in symptom management (Kluger et al., 2016; Nazarova et al., 2022; Li et al., 2023; Park et al., 2023). However, the mechanisms underlying the efficacy of these acupoint combinations warrant further investigation.

Recent studies indicate that patients with PD exhibit dysbiosis of the intestinal microbiota, leading to the production of toxic metabolites that can compromise the blood–brain barrier and transmit neurotoxic signals to the brain through the microbiota-gut-brain axis (Zheng et al., 2021; Ma et al., 2024). Specifically, one study demonstrated that electroacupuncture (EA) improves motor function and modulates the gut microbiota in PD rats, fostering the growth of beneficial bacteria while suppressing harmful ones. This modulation helps alleviate PD symptoms by decreasing inflammation and oxidative stress induced by lipopolysaccharides (LPS), potentially via the gut-microbiome-brain axis (Hu et al., 2024). Furthermore, acupuncture has been shown to not only relieve gastrointestinal disturbances but also normalize the overactivity of microglia and astrocytes in the striatum and substantia nigra, thereby reducing inflammatory responses and preventing apoptosis. Predictive functional analyses have also revealed that acupuncture can restore physiological processes related to PD pathogenesis, such as glutathione and methane metabolism (Jang et al., 2020b). These findings suggest that acupuncture may influence the gut microbiota, with metabolites derived from these microbial populations potentially diminishing nigrostriatal inflammation or oxidative stress through vagal pathways or by crossing the blood–brain barrier, ultimately enhancing motor coordination. However, these studies focus on a single pathway and fail to establish a systematic mechanistic framework through multi-omics integration analysis. This unidimensional research approach disrupts the evidence chain linking efficacy verification to mechanism interpretation, making it challenging to fully elucidate the multi-target regulatory characteristics of acupuncture therapy. Therefore, we aimed to elucidate the underlying preliminary mechanisms by incorporating a joint analysis of gut microbiota, metabolomic, and brain function networks.

However, this study has several limitations. First, while the current sample size was calculated based on UPDRS III outcomes, it is relatively small for exploratory analyses of microbiota and metabolomic results. For future mechanistic studies, we will reassess the sample size based on the findings from this trial and plan to expand it, potentially through multicenter collaborations to increase the data volume and validate the reliability of our preliminary findings. Second, the intervention period of 4 weeks and follow-up of 8 weeks are quite limited for assessing sustainable therapeutic effects in Parkinson’s disease. Consequently, the long-term impact on gut microbiota and brain function may not be adequately captured within this time frame.

## 4 Conclusion

In conclusion, our study aims to determine how acupuncture can improve the quality of life of patients with the PIGD subtype of PD through a comprehensive and multidisciplinary approach. The results of this study will contribute to the growing body of evidence supporting acupuncture as a viable adjunctive therapy, paving the way for more effective and personalized interventions for PD management.
