# Supplementary material for: Effects and mechanisms of acupuncture for PIGD-subtype Parkinson’s disease via integration of fMRI and gut microbiota-metabolomics analysis: protocol for a prospective randomized controlled trial
Source: Front Aging Neurosci. 2025 May 13;17:1534165. doi: 10.3389/fnagi.2025.1534165 (PMC12106412; doi:10.3389/fnagi.2025.1534165)
Supplement: Supplementary file 1 [file Data_Sheet_1.PDF]

China Clinical Trial Registry Registration Data Set

Data category	Information
Primary registry and trial identifying number	ChiCTR2300071703
Date of registration in primary registry	June 4th, 2023
Source(s) of monetary or material support	the Research Project of Traditional Chinese Medicine in Zhejiang Province [grant number: 2023ZL224]
Public title	Research of acupuncture on motor function in patients with PIGD Parkinson's disease based on functional magnetic resonance imaging
Countries of recruitment	China
Health condition(s) or problem(s) studied	Parkinson's disease
Intervention(s)	acupuncture
active comparator	sham acupuncture
Key inclusion and	1. A primary PD diagnosis must be confirmed by the Parkinson's Disease Society Brain Bank.<br>2. The Hoehn–Yahr level must be between 1.5 and 3.0, based on the PD grading scale.<br>3. There is no sex restriction; however, participants must be right-handed and aged between 50 and 85 years (inclusive).<br>4. According to the Uniform Parkinson's Disease Rating Scale III (UPDRS III), the PIGD criteria are met if the ratio of the mean postural instability and gait disturbance score to the tremor score is 0.90 or less.<br>5. Maintenance of the original Western medicine treatment plan for 3 months or more.

	6. The Mini-Mental State Examination (MMSE) must demonstrate that participants do not have any cognitive impairment. Scores are based on educational level: >17 for illiteracy, >20 for primary school, >22 for middle school, and >23 for university (including junior college).<br>7. Normal vision, hearing, and language abilities.<br>8. Informed consent and willing participation from participants.
Exclusion criteria	Presence of neurological diseases that significantly impact gait.<br>Diagnosis of secondary Parkinson’s syndrome or presence of cognitive impairment.<br>Severely affected by hypertension, vascular disease, cardiac insufficiency, and bone and joint systemic diseases that affect walking ability.<br>Contraindications for undergoing functional magnetic resonance imaging (fMRI).<br>Patients who have a history of alcohol or narcotic abuse or are pregnant or breastfeeding.
Study type	Interventional study
Target sample size	AC:32; CO:32
Recruitment status	Prospective registration
Primary outcome(s)	Parkinson's disease-related scales (UPDRSIII, Unified-Parkinson’s Disease Rating Scale III)
Key secondary outcomes	Berg Balance Scale; Gait analysis;<br>rs-fMRI(resting-state functional magnetic resonance imaging); intestinal flora analysis; non-targeted metabolomics analysis
