# Supplementary material for: Effects and mechanisms of acupuncture for PIGD-subtype Parkinson’s disease via integration of fMRI and gut microbiota-metabolomics analysis: protocol for a prospective randomized controlled trial
Source: Front Aging Neurosci. 2025 May 13;17:1534165. doi: 10.3389/fnagi.2025.1534165 (PMC12106412; doi:10.3389/fnagi.2025.1534165)
Supplement: Supplementary file 2 [file Data_Sheet_2.PDF]

浙江医院伦理审查委员会 临床试验审查批件

Approval Letter of Ethics Review Committee of Zhejiang Hospital

批件号 Approval NO.：2023 临审第（15K）号　　签发日期 Date of issue：2023.03.16

项目名称 Study Title	基于静息态功能磁共振观察针刺治疗对 PIGD 帕金森病患者脑功能的影响		
申办方 Sponsor	浙江医院		
主要研究者 Principal Investigator	盛吉莅	承担科室 Department	针灸科
审查类别 Category of Review	初始审查	审查方式 Type of Review	快速审查
审查日期 Date of Review	2023.03.15	审查地点 Location of Review	各自办公室
审查文件清单 Reviewed Items	1. 临床课题研究初始审查申请表<br>2. 研究方案，版本号：1.0 版，日期：2023.2.21<br>3. 立项合同书<br>4. 知情同意书，版本号：1.0 版，日期：2023.2.21<br>5. 招募广告，版本号：1.0 版，日期：2023.2.21<br>6. 主要研究者履历<br>7. 研究者责任声明<br>8. 研究者利益冲突声明<br>9. 病例报告表，版本号：1.0 版，日期：2023.2.21		
审评意见 Evaluation	本伦理审查委员会对研究者递交的上述材料进行了快速审查，认为研究者资质、临床研究方案、知情同意书、招募广告及受益与风险评估等基本符合伦理规范，同意实施临床研究。		
审查决定 Decision	委员会对该方案的审查决定为：　■批准（Approval）		
年度/定期跟踪审查 Continual Review	该研究进行过程中将接受伦理审查委员会的跟踪审查？■是(Yes)　□否(No)<br>审查频率为该研究批准之日起每 12 个月一次，首次请于 2024 年 03 月 15 日前 1 个月递交研究进展报告。<br>伦理审查委员会有根据实际进展情况改变跟踪审查频率的权利。		

注意事项：

1. 请遵循我国相关法律、法规和规章的伦理原则。
2. 请遵循经本伦理审查委员会批准的临床研究方案、知情同意书、招募材料开展本研究，保护受试者的健康与权利。对研究方案、知情同意书和招募材料等的任何修改，均须得到伦理审查委员会审查同意后方可实施。
3. 在浙江医院发生的医疗器械严重不良事件或药物可疑且非预期严重不良反应及研发期间安全性更新报告须按照 NMPA/GCP 最新要求及时递交我院伦理审查委员会，国内外其他中心发生的严重不良事件或药物可疑且非预期严重不良反应需定期汇总后递交伦理审查委员会，伦理审查委员会有权对其评估做出新的决定。
4. 自今日起，无论试验开始与否，请在跟踪审查日到期前 1 个月提交研究进展报告；若研究正在进行中，请在批件到期前 2 个月，递交研究进展报告，须得到伦理审查委员会审查同意延长批件有效期后方可继续进行。
5. 申办者应当向组长单位伦理审查委员会提交中心研究进展报告汇总；当出现任何可能显著影响试验进行或增加受试者危险的情况时，请申请人及时向伦理审查委员会提交书面报告。
6. 研究纳入了不符合纳入标准或符合排除标准的受试者，符合中止试验规定而未让受试者退出研究，给予错误治疗或剂量，给予方案禁止的合并用药等没有遵从方案开展研究的情况；或可能对受试者的权益或健康以及研究的科学性造成不良影响等违背 GCP 原则的情况，请申办者、监察员或研究者提交违背方案报告。
7. 申请人暂停或提前终止临床研究，请及时提交暂停或终止研究报告。
8. 完成临床研究，请申请人提交结题报告。
9. 凡涉及中国人类遗传资源采集标本、收集数据等研究项目，必须获得中国人类遗传资源管理办公室批准后方可在本中心开展研究。
10. 凡经我院伦理审查委员会批准的研究项目在实施前，申请人应按相关规定在国家卫健委、药审中心等的临床试验登记备案信息系统平台登记研究项目相关信息。

主任委员签字<br>Chairman Signature	[signature]
批准日期<br>Approval Date	2023.03.16
伦理审查委员会<br>Stamp of ZJEC	浙江医院伦理审查委员会（盖章） [stamp: 浙江医院伦理审查委员会]
批件有效期<br>Period of Validity	此批件的有效期为（2023.03.16-2026.03.15），逾期未实施的，自行废止。
声明<br>Statement	本伦理审查委员会的职责、人员组成、操作程序及记录遵循中华人民共和国颁布的 GCP 和 ICH-GCP 的伦理审查原则，并遵守中国的相关法律及法规。

地址：杭州市灵隐路 12 号　邮编：310013　电话：0571-81595231　联系人：谢小萍　邮箱：zjyykjkli@163.com
