# Supplementary material for: Effects and mechanisms of acupuncture for PIGD-subtype Parkinson’s disease via integration of fMRI and gut microbiota-metabolomics analysis: protocol for a prospective randomized controlled trial
Source: Front Aging Neurosci. 2025 May 13;17:1534165. doi: 10.3389/fnagi.2025.1534165 (PMC12106412; doi:10.3389/fnagi.2025.1534165)
Supplement: Supplementary file 3 [file Data_Sheet_3.PDF]

Informed Consent Form and Informed Information Page

You will be invited to participate in a clinical study based on the effects of resting-state functional magnetic resonance imaging (fMRI) observation acupuncture treatment on brain function in patients with postural instability/gait disorder (PIGD) of Parkinson's disease.

Before you decide whether to participate in the study, read the following as carefully as possible, which will help you understand the study. This study has passed the ethical review committee of Zhejiang Hospital.

Research background

Parkinson's disease is one of the most recognized refractory diseases worldwide. Patients with Parkinson's disease majorly experience movement disorders, such as static tremors, bradykinesia, increased muscle tone, and posture gait that have the characteristics of progressive disability, which can seriously affect the quality of life, causing a huge burden to the family and society. Therefore, ways to improve Parkinson's symptoms, delay the disease process, and improve the quality of life have become a medical research hotspot. Presently, the treatment of Parkinson's disease is mainly levodopa and dopamine receptor agonists. This treatment is effective and can partially relieve symptoms; however, it has many adverse reactions and can aggravate movement disorders. Therefore, a more effective treatment is urgently required; clinical trials have proven that acupuncture has a good immediate effect, no side effects, and easy acceptance by patients, which can improve the quality of life and reduce the burden on society and family.

Purpose of research

To assess the clinical effectiveness and mechanisms of acupuncture for patients with the PIGD subtype of PD.The study results will pave the way for future research.

What do you need to do if you participate in the study?

If you meet the inclusion criteria and agree to participate, the trial study will be conducted in the following steps:

You will be randomly divided into the two treatment groups, and the probability of you being divided into both groups is as high as the positive and negative probability of losing a coin.

As part of the trial preparation, we will learn more about your disease and leave your personal information with the investigators. Scale assessments and gait and brain

function tests will be conducted before and after the 4-week observation period. Additionally, the serum and stool samples will be collected for 16S rRNA sequencing and LC-MS/MS analysis to uncover mechanisms. Scale assessment and gait testing will be repeated after the 12-week follow-up period.

What are the conditions for inclusion and exclusion?Diagnostic criteria

The diagnostic criteria will adhere to the standards updated by International Movement Disorder Society in 2015. The criteria for PIGD-subtype will be met if the ratio of the mean scores for these disturbances to the tremor score is ≤0.90.

Recruitment criteria

1. Patients with primary Parkinson's disease who met the diagnostic criteria of the UK Parkinson's Brain Bank;
2. Parkinson's disease classification scale (Hoehn–Yahr) grade 1.5–3.0;
3. Sex limitation, right-handed, age 50–85 years (inclusive);
4. Visual, hearing, and language are normal;
5. Maintenance of the original Western medicine treatment plan for ≥3 months.
6. No cognitive dysfunction, as assessed by the Summary Mental Status Scale (mini-mental state examination, MMSE scores are based on educational level: >17 for illiteracy, >20 for primary school, >22 for middle school, and >23 for university);
7. Agreeing and cooperating with the trial-related work, volunteering to participate in the trial study, and signing the informed consent form.

Exclusion criteria

1. Other neurological diseases that affect gait.
2. Secondary Parkinson's syndrome or combined cognitive dysfunction.
3. Severe hypertension, vascular disease, cardiac dysfunction, and bone and joint system disease affecting walkers.
4. Pregnancy and lactation.
5. Alcohol or narcotics abuse.

What will you benefit from this study if you participate in it?

Participation in this study will help to improve motor dysfunction in patients with Parkinson's disease.

What are the risks of me participating in the study?

Acupuncture may cause dizziness, blood flow blockage, and acupuncture site bleeding. Participants may also experience some other discomfort during the trial.

Kindly tell your study physician immediately about any discomfort so that he/she can address it.

Will participating in this study increase my medical expenses?

Treatment and medical tests during the study are free and will not increase your medical costs.

What compensation will be made for participating in this study?

When you participate in this study, you will receive reimbursement for traffic compensation within city limits.

Compensation for damages

If you have any injury associated with this experimental study, our research group will compensate you based on the relevant national laws and regulations.

Is personal information confidential?

Information about your participation in this study will be recorded in the study medical chart/case report form. All test results (including personal data and laboratory documents) appearing in the original medical records will be kept completely confidential to the extent permitted by law. Your name will not appear in the case report form; only your name pinyin abbreviation and the number assigned when you entered the trial will be used. Related research summaries, articles, and public publications, if necessary, will only include your name pinyin abbreviation and number.

If necessary, the drug regulatory department, the ethics committee, or the project funding department may consult the information of the study participants. However, without permission, they will not use the details for other purposes or disclose them to other groups.

How do you get more information?

You can ask any questions about this pilot study at any time; please consult Dr. Sheng at 151××××1150.v. Your doctor will promptly notify you if there is any important new information during the trial that may affect your willingness to continue participating in the study.

Must I attend this study, or can I quit midway?

Participation in this study is entirely dependent on your willingness, and you may refuse to participate in this study. You will have the right to withdraw from the study at any time during the study. If you refuse to participate or withdraw, your benefits will not be affected and discriminated against in retaliation. However, if you

choose to participate in this study, we expect you to continue the whole trial process. Furthermore, your doctor or investigator may suspend your participation in this trial at any time for your best interest.

Are there any other treatments currently available?

Presently, drug therapy is still the most important treatment method for Parkinson's disease at home and abroad. Levodopa therapy is the gold standard for Parkinson's disease. Clinically frequently used drugs such as Ning and Medoba are the most effective drugs for treating Parkinson's disease.

What should I do right now?

You will decide whether to participate in this pilot study. You can discuss it with your family or friends before making a decision. Before you decide to participate in the trial, please ask your doctor any questions until you fully understand this trial study.

Ethics Committee

If you are dissatisfied with the study, please contact the Ethical Review Committee of Zhejiang Hospital.

Office of Ethics Committee: Office of Ethics Committee, Building 8, Lingyin Hospital, Zhejiang Hospital. Tel: 0571-81595231.

Thank you for reading the above materials. If you decide to participate in this trial study, please tell your doctor that he/she will arrange everything about the study on your behalf. Please keep this information.

Informed Consent Form, Consent Signature Page

Consent statement

1. I have read this informed consent form, and the persons responsible for the project have explained to me in detail the purpose, content, risks, and benefits of the trial.

2. I have discussed and asked relevant questions about this study, and I am satisfied with the answers to these questions.

3. I have enough time to make my decisions.

4. I voluntarily agree to participate in the clinical study described in this article.

5. If I quit due to this study, tell the doctor about the changes in time,

6. If I need to take any other treatment due to changes in my condition, I will ask the doctor for his opinion in advance or tell the doctor truthfully afterward.

7. I agree with the representative of the ethics committee or the project funding department to consult my research materials.

8. I will obtain a signed and dated consent.

Finally, I decided to participate in this pilot study and promised to follow the doctor's advice.

Signature: Date:_______________

Contact Number:_______________

Date:_____________

Telephone Number:_____________

I confirm that the details of the study, including its rights and possible benefits and risks, were explained to the participant, and I provided a copy of the signed informed consent form.

Physician Signature: Date:__________________

Study Physician Contact Information:

(This page is a necessary part of the subject's informed consent form, which must be signed and dated by the subject or the legal representative and the study doctor.）
