# Supplementary material for: Effects and mechanisms of acupuncture for PIGD-subtype Parkinson’s disease via integration of fMRI and gut microbiota-metabolomics analysis: protocol for a prospective randomized controlled trial
Source: Front Aging Neurosci. 2025 May 13;17:1534165. doi: 10.3389/fnagi.2025.1534165 (PMC12106412; doi:10.3389/fnagi.2025.1534165)
Supplement: Supplementary file 5 [file Data_Sheet_5.PDF]

UK Parkinson’s Disease Society Brain Bank clinical diagnostic criteria

Step 1 Diagnosis of Parkinsonian syndrome

- Bradykinesia (slowness of initiation of voluntary movement with progressive reduction in speed and amplitude of repetitive actions)
- And at least one of the following:
  muscular rigidity
  4-6 Hz rest tremor
  postural instability not caused by primary visual, vestibular, cerebellar, or proprioceptive dysfunction.

Step 2 Exclusion criteria for Parkinson's disease

- History of repeated strokes with stepwise progression of parkinsonian features
- History of repeated head injury
- History of definite encephalitis Oculogyric crises
- Neuroleptic treatment at onset of symptoms
- More than one affected relative
- Sustained remission
- Strictly unilateral features after 3 years
- Supranuclear gaze palsy
- Cerebellar signs
- Early severe autonomic involvement
- Early severe dementia with disturbances of memory, language, and praxis Babinski sign
- Presence of cerebral tumour or communicating hydrocephalus on CT scan
- Negative response to large doses of levodopa (if malabsorption excluded)
- MPTP exposure

Step 3 Supportive prospective positive criteria for Parkinson's disease

(Three or more required for diagnosis of definite Parkinson's disease)

- Unilateral onset
- Rest tremor present
- Progressive disorder
- Persistent asymmetry affecting side of onset most
- Excellent response (70-100%) to levodopa
- Severe levodopa-induced chorea
- Levodopa response for 5 years or more
- Clinical course of 10 years or more
