# Supplementary material for: Effects and mechanisms of acupuncture for PIGD-subtype Parkinson’s disease via integration of fMRI and gut microbiota-metabolomics analysis: protocol for a prospective randomized controlled trial
Source: Front Aging Neurosci. 2025 May 13;17:1534165. doi: 10.3389/fnagi.2025.1534165 (PMC12106412; doi:10.3389/fnagi.2025.1534165)
Supplement: Supplementary file 6 [file Data_Sheet_6.PDF]

Questions for Blinding Assessment (BTQ)

Question1：Perceived Treatment Allocation

"Do you believe you received real acupuncture, sham acupuncture, or are you unsure?"

Options: Real acupuncture / Sham acupuncture / Unsure

Question2: Certainty of Judgment

"How certain are you about your answer above?"

Options: 1 (Very uncertain) – 5 (Very certain)

Question3:Detection of Unusual Sensations

"Did you notice any unusual sensations (e.g., needle insertion, warmth) or procedures during the treatment?"

Options: Yes / No

Question4:Suspicion of Treatment Authenticity

"Was there anything that made you suspect the treatment might not be real acupuncture?"

Options: Yes (please specify) / No

Question5: Alignment with Expectations

"How well did this treatment align with your expectations of acupuncture?"

Options: Not at all – Completely

Evaluation Criteria

Blinding Success:

1) Participants complete all questions without raising doubts.
2) No reports of unusual sensations (Questions 3/4 answered "No").
3) No denial of receiving acupuncture (Question 1 not answered "Sham acupuncture" or "Unsure").

Blinding Failure:

1) Participants explicitly report unusual sensations (Questions 3/4 answered "Yes").
2) Participants deny receiving acupuncture (Question 1 answered "Sham acupuncture" with high certainty in Question 2).
